# Au nanobipyramids@mSiO_2_ core–shell nanoparticles for plasmon-enhanced singlet oxygen photooxygenations in segmented flow microreactors†‡

**DOI:** 10.1039/d0na00533a

**Published:** 2020-09-11

**Authors:** Carlos Mendoza, Anthony Désert, Denis Chateau, Cyrille Monnereau, Lhoussain Khrouz, Fréderic Lerouge, Chantal Andraud, Jean-Christophe M. Monbaliu, Stéphane Parola, Benoît Heinrichs

**Affiliations:** Nanomaterials, Catalysis & Electrochemistry (NCE), Department of Chemical Engineering, University of Liège B-4000 Liège Belgium cmendoza@uliege.be; Université de Lyon, Ecole Normale Supérieure de Lyon, CNRS, Université Lyon 1, UMR 5182, Laboratoire de Chimie 46 Allée d'Italie Lyon F69364 France; Center for Integrated Technology and Organic Synthesis (CiTOS), Research Unit MolSys, University of Liège B-4000 Liège Belgium

## Abstract

The plasmonic features of gold nanomaterials provide intriguing optical effects which can find potential applications in various fields. These effects depend strongly on the size and shape of the metal nanostructures. For instance, Au bipyramids (AuBPs) exhibit intense and well-defined plasmon resonance, easily tunable by controlling their aspect ratio, which can act synergistically with chromophores for enhancing their photophysical properties. In Rose Bengal-nanoparticle systems it is now well established that the control of the dye-to-nanoparticle distance ranging from 10 to 20 nm as well as spectral overlaps is crucial to achieve appropriate coupling between the plasmon resonance and the dye, thus affecting its ability to generate singlet oxygen (^1^O_2_). We have developed AuBPs@mSiO_2_ core–shell nanostructures that provide control over the distance between the metal surface and the photosensitizers for improving the production of ^1^O_2_ (metal-enhanced ^1^O_2_ production – ME^1^O_2_). A drastic enhancement of ^1^O_2_ generation is evidenced for the resulting AuBPs and AuBPs@mSiO_2_ in the presence of Rose Bengal, using a combination of three indirect methods of ^1^O_2_ detection, namely *in operando* Electron Paramagnetic Resonance (EPR) with 2,2,6,6-tetramethylpiperidine (TEMP) as a chemical trap, photooxygenation of the fluorescence probe anthracene-9,10-dipropionic acid (ADPA), and photooxygenation of methionine to methionine sulfoxide in a segmented flow microreactor.

## Abstract 2

Gold nanobipyramids coated with 12 nm mSiO_2_ shells exhibited metal-enhanced ^1^O_2_ generation in continuous-flow photooxygenation reactions.

## Graphical abstract

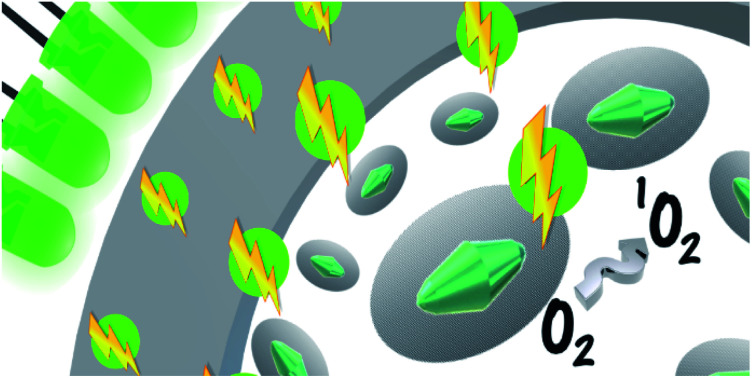

## 1. Introduction

Plasmonic noble metal-based nanostructures have received intensive attention during the last decade owing to the integration of plasmonic functionality with other physicochemical properties, such as catalytic activity,^1,2^ optical imaging,^3,4^ and photodynamic/photothermal cancer therapies.^5,6^ Gold nanoparticles (AuNPs) possess Localized Surface Plasmon Resonance (LSPR), which arises from the collective oscillation of conduction electrons in response to incident light under resonance conditions. The size and geometry of Au NPs can be controlled during their colloidal synthesis by varying the experimental parameters in order to obtain various shapes such as spheres,^7^ triangles,^8,9^ rods,^10–14^ stars^15^ or bipyramids.^16–18^ These anisotropic gold bipyramids (AuBPs) possess a Transverse LSPR (T-LSPR) and an intense Longitudinal LSPR (L-LSPR) depending on the nanostructure dimensions and aspect ratio. We have already shown the controlled synthesis of AuBPs with L-LSPR from 600 nm to the NIR region^16^ and surface modification for preparing hybrid materials.^19^ We have also demonstrated the potential of such AuBPs for plasmon enhancement effects in various systems: increase of the fluorescence emission and photoresistance of coupled dyes,^18^ improvement of nonlinear absorption of hybrid materials,^20^ or enhancement of photocatalytic activity for TiO_2_/SiO_2_ thin films.^21^ In the present work, AuBPs have been investigated for photocatalysis *via*^1^O_2_ production.

The transfer from the batch mode to continuous-flow mode in order to improve light–matter interactions on smaller length scales has proved that ^1^O_2_ photogeneration benefits from “going to flow”.^22^ Flow photochemistry has shown numerous potential benefits for this kind of photochemical reaction, such as high light penetration, improved O_2_ transfer to the liquid phase, ease of processing multiphase reaction mixtures, and potential to include inline analytical technologies.^22–28^ The most common method to generate ^1^O_2_ involves activation of ground state triplet oxygen (^3^O_2_), through energy transfer from a light activated photosensitizer (PS). Rose Bengal (RB) shows absorption bands between 480 and 550 nm, and is renowned for its high quantum yield (*ϕ*Δ = 0.76) for the generation of ^1^O_2_.^28,29^ The photophysical properties of RB in close proximity to metal NPs are strongly affected by the plasmon coupling near the metallic surfaces, which may lead to an increase in fluorescence emission.^30–32^ In addition to such Metal Enhanced Fluorescence (MEF), Metal Enhanced Phosphorescence (MEP) and ME^1^O_2_ have also been previously described by Geddes *et al.*^33,34^ In RB–AuNP systems, the control of the dye-to-nanoparticle distance is crucial to achieve cooperative coupling between the plasmon resonance and the dye. More precisely, RB reportedly needs to be located at a distance of about 10 nm to achieve maximal enhancement for both fluorescence^35^ and ^1^O_2_ generation efficiencies.^36^

Typically, ^1^O_2_ can be revealed directly by measuring ^1^O_2_ phosphorescence at 1270 nm or indirectly, by using chemical probes that react with ^1^O_2_ producing compounds that are the detected species. In previous studies, we have studied homogeneous and heterogeneous ^1^O_2_ generation by indirect methods.^28^ In this work, the obtained anisotropic AuBPs@mSiO_2_ nanostructures with SiO_2_ thicknesses of 6 and 12 nm were completely characterized in terms of morphology, and spectroscopic and photophysical features. AuBPs@mSiO_2_ with 12 nm showed higher stability and they were chosen for ^1^O_2_ generation tests. Their impact on the ^1^O_2_ production by RB in solution was evaluated simultaneously by three complementary protocols for indirect ^1^O_2_ detection: *in operando* Electron Paramagnetic Resonance with 2,2,6,6-tetramethylpiperidine (EPR/TEMP), photooxygenation of anthracene-9,10-dipropionic acid (ADPA), and photooxygenation of methionine (Met) into methionine sulfoxide (MetO) using a segmented flow microreactor.

## 2. Results and discussion

### Characterization of AuBPs and AuBPs@mSiO_2_ core–shell NPs

**Fig. 1:**
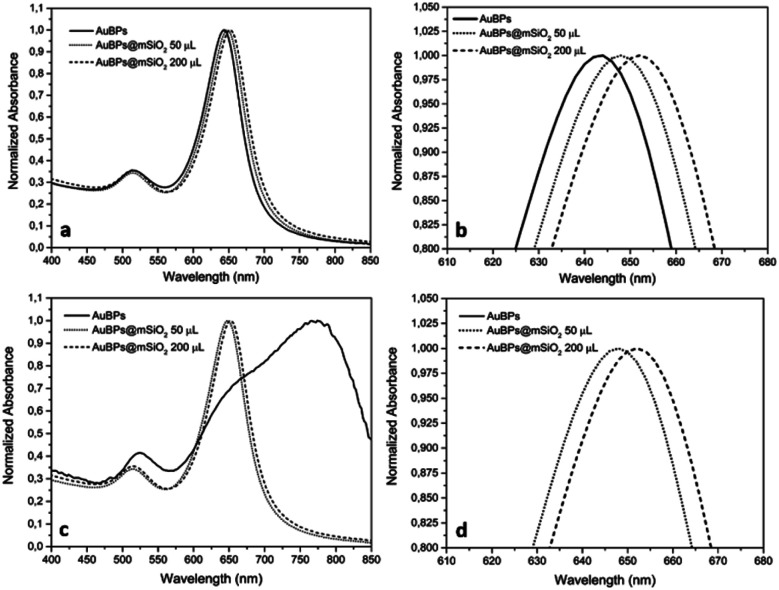
UV-vis spectra of AuBPs and AuBPs@mSiO_2_ core–shell NPs prepared with 50 and 200 μL of TEOS and then dispersed in (a) H_2_O, (b) H_2_O zoomed, (c) EtOH, and (d) EtOH zoomed.

Highly truncated AuBPs, synthesized in previous studies,^16,20,21^ were employed as substrates for the growth of mesoporous SiO_2_ shells with two different thicknesses to obtain a stable physical separation between the metallic surface and the PS. Surface modifications of Au nanostructures may affect their optical properties. It is well known that the presence of SiO_2_ shells produces a red-shift in the LSPR of the metal nanoparticle in such core–shell nanostructures. AuBPs@mSiO_2_ NPs prepared with 50 and 200 μL of TEOS were characterized by UV-vis spectroscopy. The absorbance of the AuBPs and AuBPs@mSiO_2_ NPs dispersed in H_2_O and EtOH was measured between 400 and 800 nm (Fig. 1). The UV-vis spectrum of AuBPs suspended in water presented a narrow peak at 643 nm corresponding to the plasmon resonance. AuBPs@mSiO_2_ prepared with 50 μL of TEOS presented a plasmon resonance shifted to 648 nm, while it was shifted to 652 nm for the core–shell NPs prepared with 200 μL of TEOS. Additionally, the positive surface charge resulting from adsorbed CTA^+^ cations is observed on the AuBPs and AuBPs@mSiO_2_ NPs by zeta potential measurements (Table 1). The initial AuBPs and the obtained AuBPs@mSiO_2_ core–shell NPs were then dispersed in EtOH to verify the effect of SiO_2_. AuBPs showed a broad peak shifted from 643 to >775 nm, which is characteristic of the aggregation of AuNPs, while all the synthesized AuBPs@mSiO_2_ nanostructures did not show a change the position of the plasmon resonance when they were dispersed in EtOH. This slight red shift in both solvents indicates the presence of a SiO_2_ layer around the AuBPs and the possibility of dispersion of the core–shell NPs in EtOH demonstrated the protection of the initial AuBPs from aggregation.

**Table 1:** Characterization results of AuBPs and AuBPs@mSiO_2_ core–shell NPs

	T-LSPR (nm)	L-LSPR (nm)	T-LSPR (nm)	L-LSPR (nm)	Size DLS (nm)	Zeta potential (mV)
Solvent	H_2_O		EtOH		H_2_O	
AuBPs	514	643	524	Unstable	30	24.0
AuBPs@mSiO_2_ 50TEOS	514	648	514	648	45	40.2
AuBPs@mSiO_2_ 200TEOS	514	652	515	652	75	44.8

**Fig. 2:**
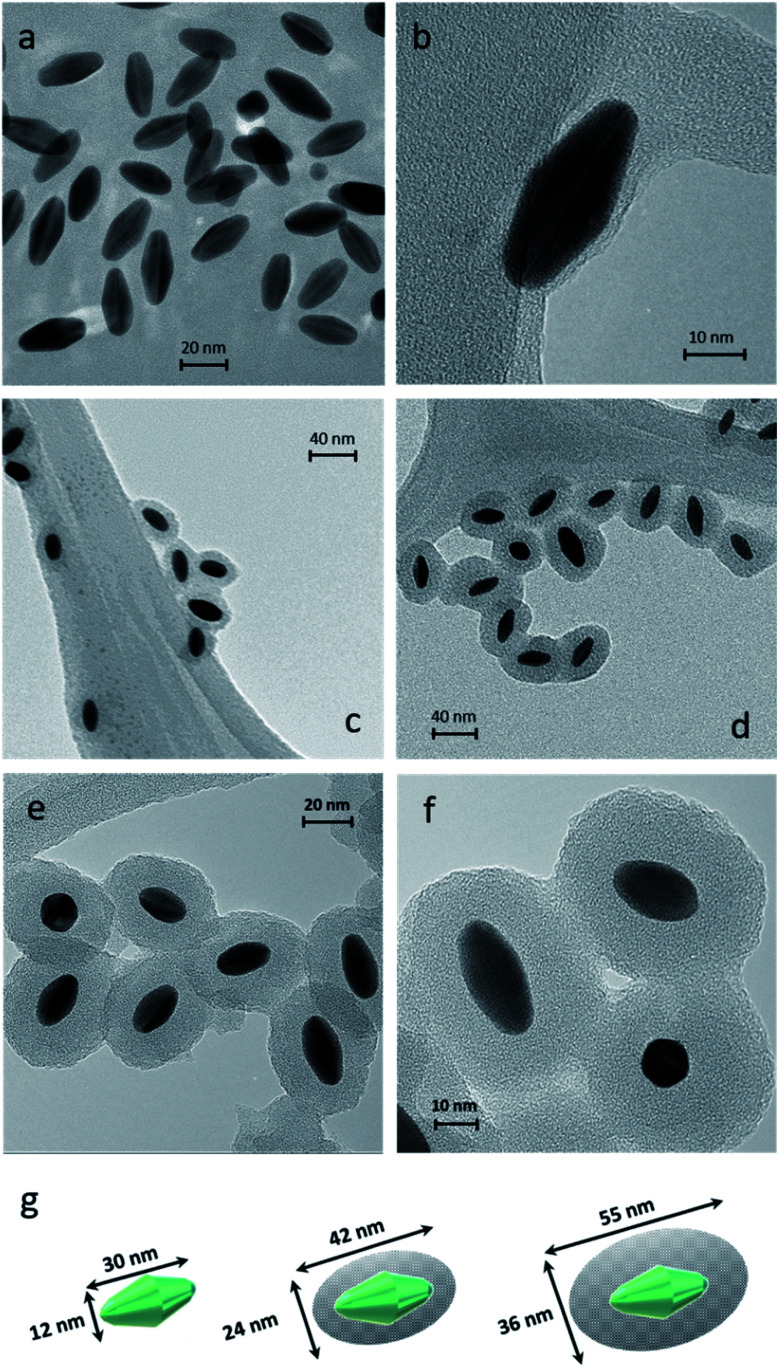
TEM images of AuBPs at a magnification of (a) 280k× and (b) 700k×, AuBPs@mSiO_2_ (c) 50TEOS and (d) 200TEOS at a magnification of 150k×, AuBPs@mSiO_2_ 200TEOS with a SiO_2_ thickness of 12 nm at magnification of (e) 280k× and (f) 490k× and (g) size calculations of AuBPs, AuBPs@mSiO_2_ 50TEOS and AuBPs@mSiO_2_ 200TEOS from images (±2.1 nm from 200 particles/20 TEM images).

Dynamic Light Scattering (DLS) is a simple and accessible technology commonly used to measure the size of nanomaterials with spherical shapes. However, this tool needs to be carefully used when measuring anisotropic nanoparticles. In our case, DLS allowed appropriate measurements of the anisotropic sample. DLS size measurements exhibited one population (monomodal) of stable NPs with hydrodynamic diameters of 30, 45, and 75 nm for AuBPs, AuBPs@mSiO_2_ obtained with 50 μL of TEOS and AuBPs@mSiO_2_ with 200 μL, respectively (see the ESI‡). Transmission electron microscopy (TEM) was employed to confirm the size of the AuBPs@mSiO_2_ core–shells and evaluate the thickness of mSiO_2_ shells (Fig. 2).

The effect of the increasing amount of TEOS is shown in Fig. 2b–d that corresponds to the samples AuBPs, AuBPs@mSiO_2_ 50TEOS and AuBPs@mSiO_2_ 200TEOS. More specifically, AuBPs@mSiO_2_ 200TEOS exhibited homogeneous mSiO_2_ shells with a thickness of 12 nm (±2.1 nm) (Fig. 2e and f). Fig. 2g shows the dimensions of AuBPs and AuBPs@mSiO_2_ core–shells calculated from TEM images (average values obtained from 200 particles/20 TEM images). The length increased from 30 to 55 nm and the width also increased from 12 to 36 nm with a final surface ratio Au/mSiO_2_ of (18/82) particle size distribution charts are shown in the ESI.‡ Furthermore, the quality of the encapsulation process was confirmed by UV-vis absorption. The spectra of AuBPs@mSiO_2_ NPs were slightly redshifted and no peak broadening was observed. These NPs were stable even in ethanol (see LSPR values in Table 1 and Fig. 6). Concordant with those results, DLS size measurements exhibited one population of stable NPs with a hydrodynamic diameter of 75 nm (see the ESI‡). Finally, the positive surface charge resulting from the adsorbed CTA^+^ cations is observed on the AuBPs and AuBPs@mSiO_2_ nanoparticles by zeta potential measurements. This is assumed to promote proximity interactions with the negatively charged RB by simple electrostatic attraction. Table 1 summarizes the characterization results of AuBPs and AuBPs@mSiO_2_ core–shell nanostructures. AuBPs@mSiO_2_ 200TEOS core–shell NPs were chosen to carry out ^1^O_2_ generation tests in combination with RB solutions due to their high stability.

### ^1^O_2_ generation by *in operando* EPR/TEMP

Singlet oxygen reacts with TEMP to produce 2,2,6,6-tetramethylpiperidine-1-oxyl radical (TEMPO) which can be observed by EPR spectroscopy.^37–39^^1^O_2_ generation was evaluated under white light irradiation (400–800 nm) on the investigated RB/AuBPs@mSiO_2_ mixtures as well as on different reference samples in order to evaluate (i) the effect of the plasmon resonance and (ii) distance-tuning between RB and AuBPs. First, EPR was carried out with the photosensitizer RB in an aqueous solution (0.045 mM) (Fig. S4a‡). AuBPs and AuBPs@mSiO_2_ in water were also measured individually as a second and third reference (Fig. S4b and c‡). Finally, AuBPs and AuBPs@mSiO_2_ in the presence of RB (0.045 mM) were measured in order to estimate the effect of the mSiO_2_ shell (Fig. S4d and e‡).

**Fig. 3:**
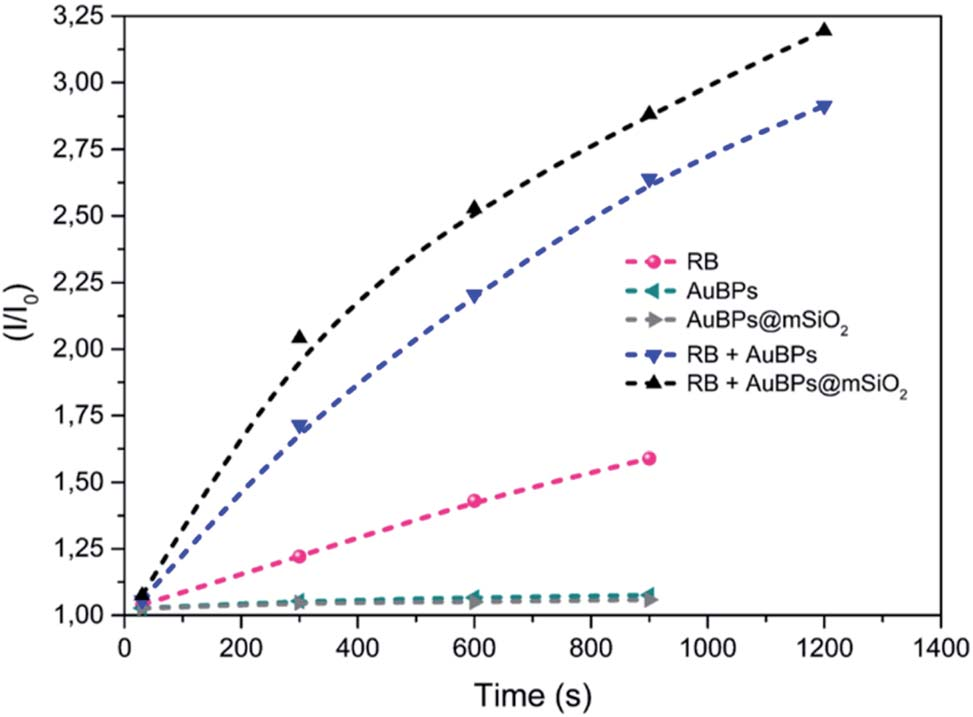
EPR relative intensities of photoinduced generation of TEMPO under visible irradiation for the different systems.

In all cases, a characteristic signal of a TEMPO radical was present in the sample even in the absence of irradiation, characteristic of a spontaneous oxidation of TEMP by addition of oxygen to generate a TEMPO derivative. This signal is not characteristic of a specific PS mediated photoinduced generation of singlet oxygen, but rather to a contamination of the TEMP sample with TEMPO. However, while no significant evolution of the system was monitored in the absence of a PS or with AuNPs alone, the RB containing sample showed a marked increase of the paramagnetic TEMPO related signal with time unambiguously indicating photosensitized ^1^O_2_ production. More significantly, a higher turn-over frequency of ^1^O_2_ was observed when RB was mixed with AuBPs in good agreement with a metal enhanced singlet oxygen generation mechanism. Finally, RB mixed with AuBPs@mSiO_2_ (Fig. S4e‡) showed higher EPR intensities than the sample which contained RB mixed with AuBPs (Fig. S4d‡). This increase in EPR intensities indicated that the presence of a 12 nm SiO_2_ shell enhances the ^1^O_2_ production of the photosensitizer containing free RB. Additionally, relative intensities (*I*/*I*_0_) were calculated from the EPR spectra and kinetics of TEMPO generation were also increased when we introduced the SiO_2_ shell, as shown in Fig. 3. An effect of saturation with the irradiation time was observed in all cases.

### ^1^O_2_ production by oxidation of ADPA

**Fig. 4:**
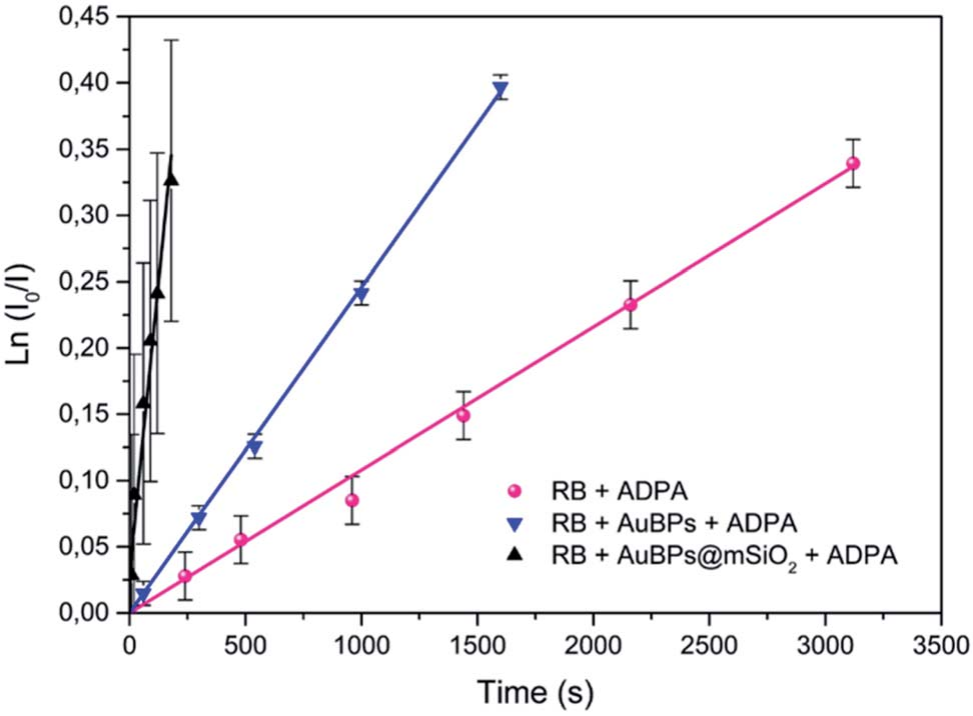
First-order kinetics linearization of the oxidation of ADPA for RB, RB in the presence of AuBPs and RB in the presence of AuBPs@mSiO_2_.

In the next step, we studied the kinetics of singlet oxygen generation using spectrofluorometry. More precisely ADPA was used as a fluorescent singlet oxygen scavenger. In the presence of singlet oxygen, monitored fluorescence is expected to undergo a first order decrease (see the ESI‡), with a kinetic constant that is proportional to the singlet oxygen generation efficiency of the studied PS. Here the PS systems consisted of RB, AuBPs in the presence of RB or AuBPs@mSiO_2_ in the presence of RB. In all cases the same concentration of RB and NPs was used. AuBPs and AuBPs@mSiO_2_ in the absence of RB were also measured as references. However, in good agreement with aforementioned EPR results, these samples did not provide a significant variation of the intensity with time. As shown in Fig. 4, ln (*I*_0_/*I*) (where *I* stands for the measured luminescence of ADPA after a given irradiation time *t*, and *I*_0_ corresponds to luminescence intensity at irradiation time *t* = 0) was plotted *vs.* the irradiation time. ADPA oxidation kinetics were then obtained from the linear fit of the experimental data (see the ESI‡ for details about the kinetic model considered).

The initial rate of RB, RB/AuBPs and RB/AuBPs@mSiO_2_ was 1.08 × 10^−4^ s^−1^, 2.46 × 10^−4^ s^−1^ and 16.2 × 10^−4^ s^−1^, respectively, with a correlation coefficient higher than 0.99 in all cases. It indicates an enhancement in the initial rate of ^1^O_2_ production of RB in the presence of AuBPs, which is even greater with the introduction of the 12 nm SiO_2_ shell for AuBPs@SiO_2_, maximizing plasmon induced photophysical effects by positioning the PS at an optimal distance from the gold core.

### ^1^O_2_ photooxygenations in a microfluidic reactor

**Fig. 5:**
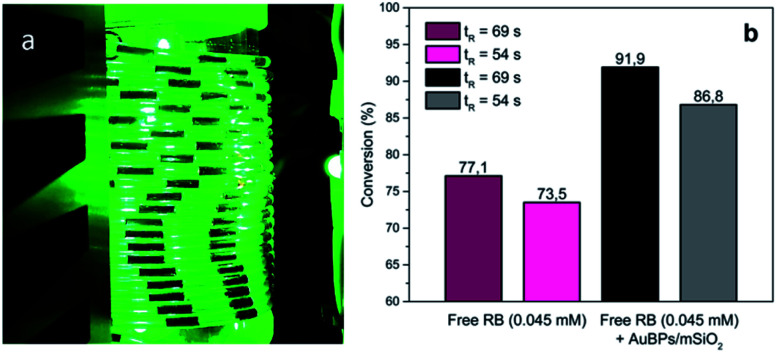
(a) G/L segmented flow, (b) conversion of Met into MetO with RB (0.045 mM) and AuBPs@mSiO_2_ in the presence of RB (0.045 mM) at a liquid flow rate of 0.5 (*t*_R_ = 69 s) and 1 mL min^−1^ (*t*_R_ = 54 s).

Building upon these promising results, the photooxygenation of Met using AuBPs@mSiO_2_ core–shell NPs in the presence of RB was attempted under segmented flow conditions in a microfluidic setup constructed from PFA capillaries and using green LEDs as a light source to benefit the various advantages offered by the microsystem technologies.^40^ The performance of the reaction with AuBPs@mSiO_2_ NPs was compared with that under the pure RB homogeneous conditions using unsupported RB and the conversion of Met was monitored by ^1^H NMR. Fig. 5 shows the segmented flow with the proximity of the green LEDs and the conversion obtained with the pure RB system and RB + AuBPs@mSiO_2_ system for different residence times.

Regarding the conversion of Met to MetO, 77.1 and 73.5% were obtained using free RB for residence times of 69 and 54 s, respectively. With AuBPs@mSiO_2_ core–shell NPs in the presence of a similar concentration of RB (0.045 mM), a higher efficiency of substrate conversion was observed under the same microfluidic conditions, which we correlate with the previously monitored metal-enhanced generation of ^1^O_2_. Namely, conversion was increased to 91.9 and 86.8% for residence times of 69 and 54 s, respectively. Additionally, UV-vis absorption spectra showed a decrease in the RB photodegradation when the PS is combined with AuBPs@mSiO_2_ NPs; while at short *t*_R_ (54 s), the difference in the RB photodegradation is marginal, RB degradation can be decreased from 48.2 to 33.5% in the presence of AuBPs@mSiO_2_ NPs for a longer *t*_R_ (69 s) (see the ESI‡). This revealed that the AuBPs@mSiO_2_ can protect the PS against oxidation and photobleaching presumably through more efficient and thus faster energy transfer from RB to surrounding molecular oxygen.

**Fig. 6:**
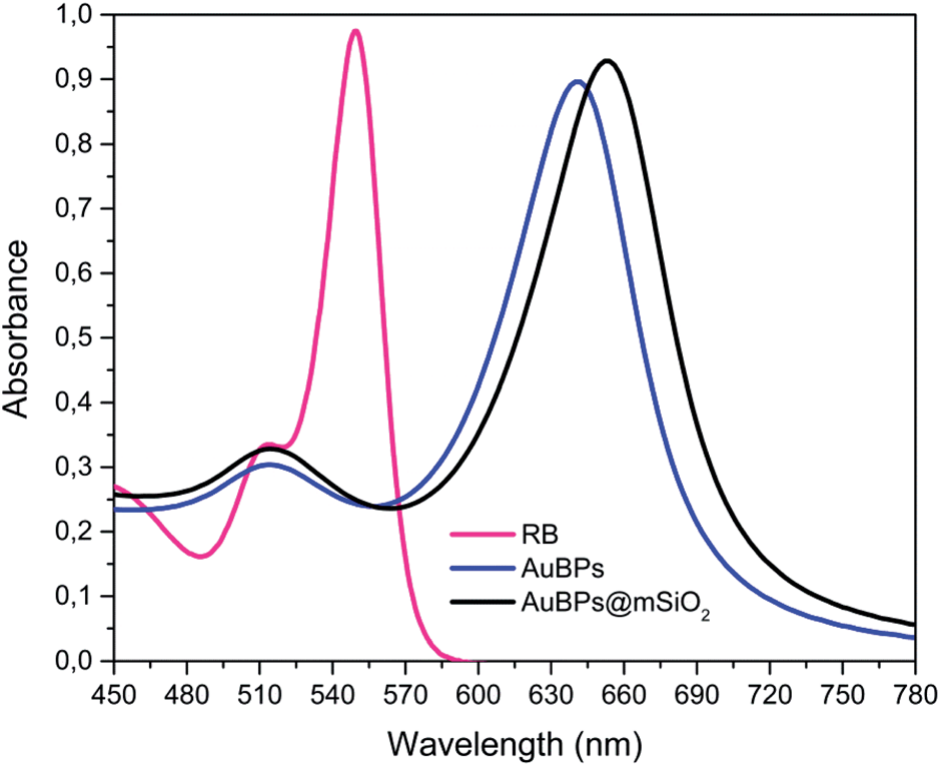
Spectral overlap of AuBPs, AuBPs@mSiO_2_ and RB.

The results obtained through the three quantitative methods clearly demonstrate an enhancement of ^1^O_2_ photogeneration *via* RB in the presence of AuBPs, which we attribute to the coupling effect between the T-LSPR of AuBPs (*λ*_T-LSPR_ ≈ 515 nm) and RB dipole (*λ*_abs_ ≈ 540–550 nm). This increases the excitation and photostability of RB molecules under green irradiation (*λ*_light_ ≈ 540–560 nm) (because of the spectral overlap between RB absorption and AuBP plasmon resonance, see Fig. 6). It should be noted that the L-LSPR could also be excited, which is the case for EPR experiments with the 400–800 nm irradiation *via* a halogen lamp. Finally, the significant improvements obtained with AuBPs@mSiO_2_ NPs, compared to AuBPs, demonstrated the importance of tuning the distance between the metal surface and the molecules for optimizing plasmon enhancement mechanisms, but also the importance of controlling the size and shape of AuNPs to combine them with the spectrum of a specific photosensitizer.^6,12,41,42^

EPR is a spectroscopic measurement technique, and thus, direct (even using a scavenger) to detect singlet oxygen. It gives us a characteristic fingerprint of its presence. The measurements of photooxygenation of substrates such as ADPA or Met are evaluation of the oxidizing nature of the photosensitized medium, without specific information on the nature of the oxidant species. The advantage of testing ADPA disappearance is the ability to follow ^1^O_2_ kinetics in a simple *in operando* manner at short times and low concentrations. This technique can be carried out with controlled irradiations. Finally, photooxygenation of Met with a synthetic purpose demonstrated the goal of the catalysts though their application. The purpose of previous tests was to detect the presence of ^1^O_2_ and its behaviour during the irradiation time, while the objective of the last test was to validate the interest of the method.

## 3. Experimental

### Materials and methods

HCl (37%), ethanol (99.8%), hexadecyltrimethylammonium bromide (CTAB, 98%), tetraethyl orthosilicate (TEOS, 99%), NaOH (98%), Rose Bengal (RB, 95%), TEMP (95%) and TEMPO (98%) were purchased from Sigma Aldrich and used as received. HAuCl_4_·3H_2_O (99.9%) and silver nitrate were purchased from Alfa Aesar, and ADPA (>98%) was obtained from Chemodex and l-methionine (Met, >99%) from TCI.

### Synthesis of AuBPs@mSiO_2_ core–shell NPs

Preparation of AuBPs using a seed mediated process has been fully described previously.^16^ In this work, a method adapted from Chateau *et al.*^16^ was utilized to prepare a concentrated ([Au] = 3.125 mM) suspension of truncated bipyramids in water with a longitudinal L-LSPR localized at 634 nm and a transverse T-LSPR at 512 nm.^43^ The concentration of CTAB was previously optimized (2.5 mM) to obtain clearly defined core–shell NPs, avoiding both the formation of AuBP clusters inside of mSiO_2_ or the homogeneous nucleation of individual mesoporous SiO_2_ NPs. 20 μL of 0.1 M CTAB solution and 10 μL of 0.1 M NaOH solution were added to 1 mL of a diluted suspension (1/5) of stock AuBPs ([Au] = 0.625 mM). After that, different amounts of TEOS were added to control the thickness of SiO_2_. 50 μL of TEOS/ethanol (1.75 wt%) solution was injected dropwise under stirring to form the first layer of the mesoporous silica shell (AuBPs@mSiO_2_ 50TEOS) and then 150 μL to obtain a second layer with a 3 h interval between additions (AuBPs@mSiO_2_ 200TEOS). The higher concentrations of TEOS did not lead to the formation of SiO_2_ shells (individual SiO_2_ spheres were observed). The reaction was conducted for 24 h, and the resultant colloidal suspension was centrifuged at 10 000 rpm for 30 min and washed 3 times with 10 μL of HCl (wt 37%) to remove the maximum amount of unreacted TEOS and the maximum amount of CTAB from the pores.

### Characterization

The dispersion and stability of AuBPs and AuBPs@mSiO_2_ were evaluated as a function of zeta potential by laser doppler velocimetry and their size was measured by DLS using a Malvern Zetasizer Nano for both measurements. The absorption spectra were recorded with a PerkinElmer UV-vis-NIR Lambda 750 spectrometer. TEM images were acquired using an FEI Tecnai G2 20 TWIN instrument operating at 200 kV, which enabled more specific size and thickness calculations of AuBPs and AuBPs@mSiO_2_.

### Singlet oxygen generation

**Fig. 7:**
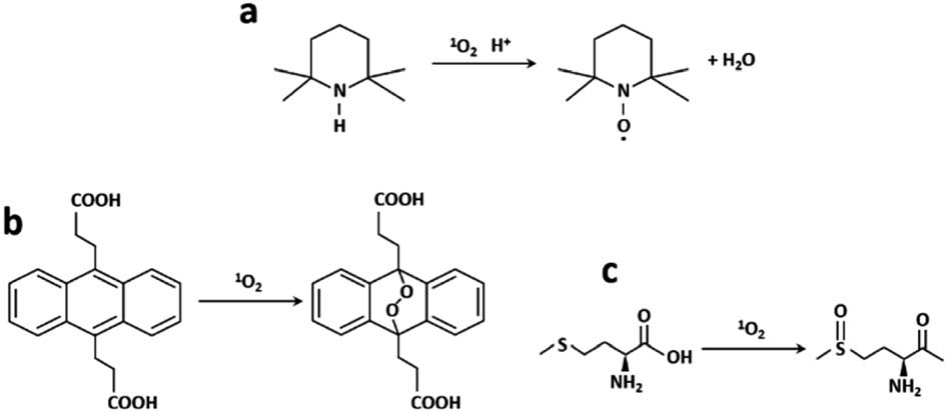
(a) TEMP to paramagnetic TEMPO, (b) ADPA to ADPA–O_2_ reaction in the presence of ^1^O_2_ and (c) ^1^O_2_ photooxygenation of Met into MetO.

The ability of generation of singlet oxygen was evaluated with three different methods: EPR/TEMP, oxidation of ADPA and photooxygenation of Met in a microreactor (Fig. 7).

### EPR/TEMP

Photogenerated ^1^O_2_ reacts with TEMP leading to the paramagnetic radical TEMPO which has a characteristic three-line EPR spectrum (Fig. 7a). RB, RB/AuBPs and RB/AuBPs@mSiO_2_ solutions ([RB] = 0.045 mM and [Au] = 0.065 mM) were irradiated with a halogen lamp using a filter which allowed obtaining a wavelength range between 400 and 800 nm. The samples were measured under normal conditions, with 100 kHz magnetic field modulation, 22 mW microwave power and 1.0 G modulation amplitude in a Bruker EMX Plus spectrometer, at room temperature. ^1^O_2_ generation was evaluated through double integration of the spectra obtaining *I* at time *t*.

### ADPA method

Polycyclic aromatic hydrocarbons such as anthracenes or rubrene are commonly used as ^1^O_2_ traps.^44^ Among them, ADPA, which can be monitored by both absorption and/or fluorescence spectroscopies,^45–48^ is highly reactive against ^1^O_2_ (reactive rate constant, *k*_r_ = 8 × 10^7^ M^−1^ s^−1^ in heavy water).^49^ ADPA shows structured absorption and fluorescence spectra with maximums around 380 nm and 430 nm, respectively. Upon reaction with ^1^O_2_, its characteristic absorption/fluorescence is bleached concomitant with the formation of an endoperoxide adduct (Fig. 7b).^49^ In this paper, the changes in the fluorescence intensity of ADPA were used to probe the kinetics of endoperoxide formation and thus to assess ^1^O_2_ generation efficiency, using a modified recently reported protocol.^50^

In all cases, freshly prepared solutions of ADPA and of the studied photosensitizer (Rose Bengal) were placed in a 3 mL quartz cuvette (1 × 1 × 3 cm), and stirred at 60 rpm for the whole irradiation period. The absorbance of the photosensitizer was set constant in all experiments (optical density of 0.3 at 378 nm) corresponding to a concentration of Rose Bengal of 0.5 μM. The initial absorbance of ADPA was of 0.1 and the Au concentration used was 0.065 mM. All samples were then irradiated using a spectrofluorometer xenon arc lamp as an irradiation source. The irradiation wavelength was centred at 540 nm, and entrance slits were set at 7 nm opening. RB luminescence was monitored at 600 nm over the whole irradiation time, to ensure that photobleaching of the latter could be considered negligible during the experiment. This setup ensured a constant irradiation power throughout all the irradiation experiments.

Conversion of the ADPA scavenger upon reaction with the photogenerated oxygen was followed by regular measurements of the luminescence signal of the latter (considering it to be linearly proportional to its concentration, which is valid in the investigated range of absorbance) consecutive to an excitation performed at 348 nm.

### Segmented flow photooxygenation of methionine

The segmented flow setup for the ^1^O_2_ photooxygenation of Met into MetO (Fig. 7c) was constructed from high purity PFA capillaries (800 μm internal diameter) wrapped in coils around a glass cylinder. The PFA coils were surrounded by 4 adjustable heat-sink integrated pillars each supporting 3 high power green LEDs (540 nm, LZ1-00G102, Led Engin) (Fig. S6‡). A Chemyx Nexus 6000 syringe pump was used to handle the suspensions loaded into a 20 mL 316 stainless steel Chemyx syringe. Two different aqueous suspensions were prepared. A first feed solution contained [Met] = 0.1 M and [RB] = 0.045 mM. The second suspension was prepared with [Met] = 0.1 M, [RB] = 0.045 mM and 1 mL of the AuBPs@mSiO_2_ NP suspension. Oxygen (Air Liquide, Alphagaz 1) was delivered at 10 mL min^−1^ with a Bronkhorst F210CTM mass flow controller. Liquid flow rates were set at 0.5 and 1 mL min^−1^, while the flow rate of gas was kept constant (12 mL min^−1^) during all the experiments. Liquid and gas inlets were then connected by a static mixer (T-Mixer, IDEX-Upchurch, natural PEEK 1/4-28 thread for 1/16′′ o.d. tubing, 0.02′′ through hole) to obtain a segmented flow. A Zaiput Flow Technologies dome-type back-pressure regulator (BPR) was inserted downstream and connected to a cylinder of compressed argon (set point: 8.5 bar). The reaction was monitored by off-line ^1^H NMR carried out on a Bruker Avance III 400 MHz spectrometer (see the ESI for details‡).

## 4. Conclusions

This paper reports the synthesis of AuBPs@mSiO_2_ core–shell NPs from previously reported AuBPs, and the ability of the photosensitizer, RB, to enhance ^1^O_2_ generation in the presence of AuBPs and AuBPs@mSiO_2_ core–shell NPs. The SiO_2_ thickness was previously adjusted with different concentrations of TEOS in order to obtain mSiO_2_ shells of around 12 nm. ^1^O_2_ production of a RB PS was measured with three different methods (EPR, the ADPA method and segmented flow photooxygenation of Met) that were consistent in showing that the generation was significantly enhanced in all cases in the presence of NPs, especially when the distance between AuBPs and RB was tuned through introduction of a 12 nm mesoporous SiO_2_ shell. Although this distance could be slightly lower due to the porous structure of SiO_2_, the exact mechanism of metal-enhanced ^1^O_2_ generation is under investigation and all these results seem to point out that ^1^O_2_ generation enhancement results from plasmon mediated promotion of PS absorption, intersystem crossing and triplet state generation. These results demonstrated the possibility of drastically improving the performance of the photosensitizer Rose Bengal by combining it with AuBPs@mSiO_2_ core–shell NPs, which may find relevant interesting applications in several fields such as fluorescence imaging (O_2_ sensing), PDT or ^1^O_2_ mediated photoreactions. Segmented flow photooxygenation in microfluidic reactors introduced herein as an applicative example opens the route toward the elaboration of a new generation of devices based on plasmonic and anisotropic core–shell NPs in combination with photosensitizers for improved synthetic oxidative photochemistry.

## Conflicts of interest

There are no conflicts to declare.

## Supplementary Material

NA-002-D0NA00533A-s001
