# Supplementary material for: Au nanobipyramids@mSiO2 core–shell nanoparticles for plasmon-enhanced singlet oxygen photooxygenations in segmented flow microreactors
Source: Nanoscale Adv. 2020 Sep 11;2(11):5280–7. doi: 10.1039/d0na00533a (PMC9416853; doi:10.1039/d0na00533a)
Supplement: NA-002-D0NA00533A-s001 [file NA-002-D0NA00533A-s001.pdf]

Electronic Supplementary Material (ESI) for Nanoscale Advances.
This journal is © The Royal Society of Chemistry 2020

Supporting Information

Au Nanobipyramids@mSiO_2 Core-Shell Nanoparticles for Plasmon-Enhanced Singlet Oxygen Photooxygenations in Continuous-Flow Microreactors

Carlos Mendoza[1,], Anthony Désert[2], Denis Chateau[2], Cyrille Monnereau[2], Lhoussain Khrouz[2], Fréderic Lerouge[2], Chantal Andraud[2], Jean-Christophe M. Monbaliu[3], Stephane Parola[2] and Benoît Heinrichs[1]*

1 Nanomaterials, Catalysis & Electrochemistry (NCE). Department of Chemical Engineering. University of Liège, B-4000 Liège, Belgium

2 Université de Lyon, Ecole Normale Supérieure de Lyon, CNRS, Université Lyon 1, UMR 5182, Laboratoire de Chimie, 46 allée d'Italie, F69364, Lyon, France

3 Center for Integrated Technology and Organic Synthesis (CiTOS). Department of Chemistry. University of Liège, B-4000 Liège, Belgium

1. Characterization of AuBPs and AuBPs@mSiO$_2$ NPs

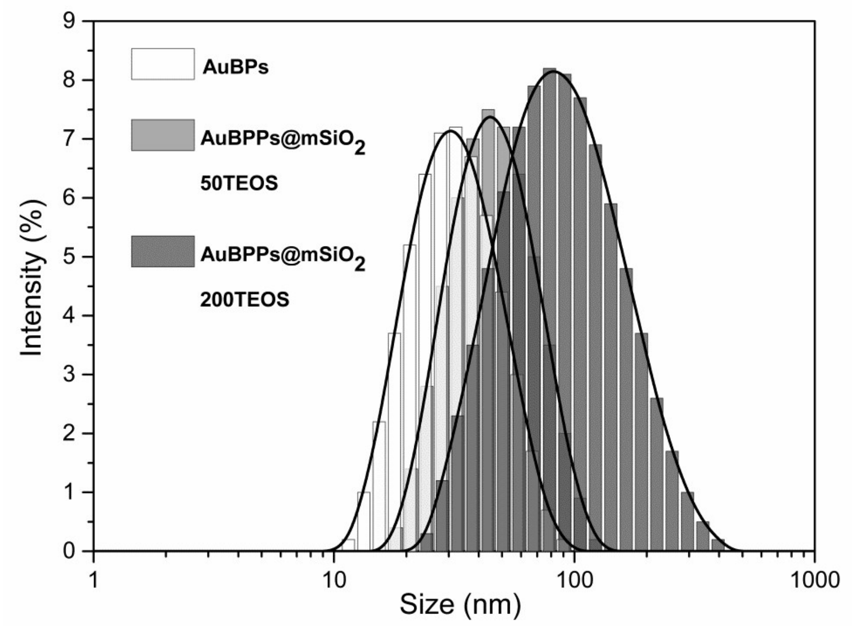

Fig. S1. DLS size measurement of AuBPs and AuBPs@mSiO$_2$ core-shell NPs

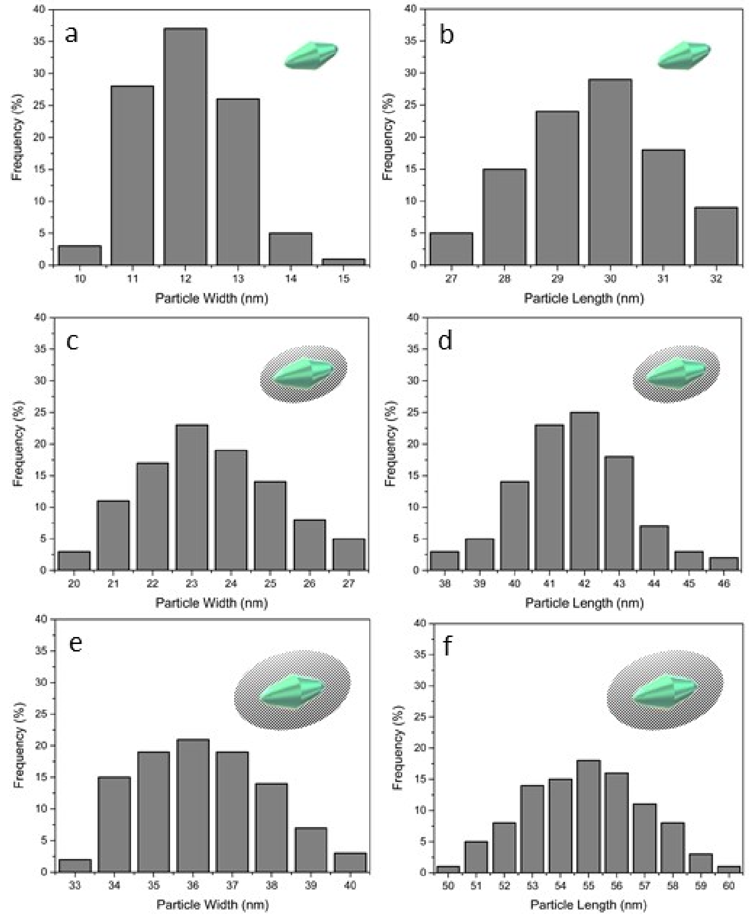

Fig. S2. Particle size distribution charts of AuBPs and AuBPs@mSiO$_2$ core-shell NPs. 200 particles were counted.

2. $^1O_2$ generation by EPR/TEMP

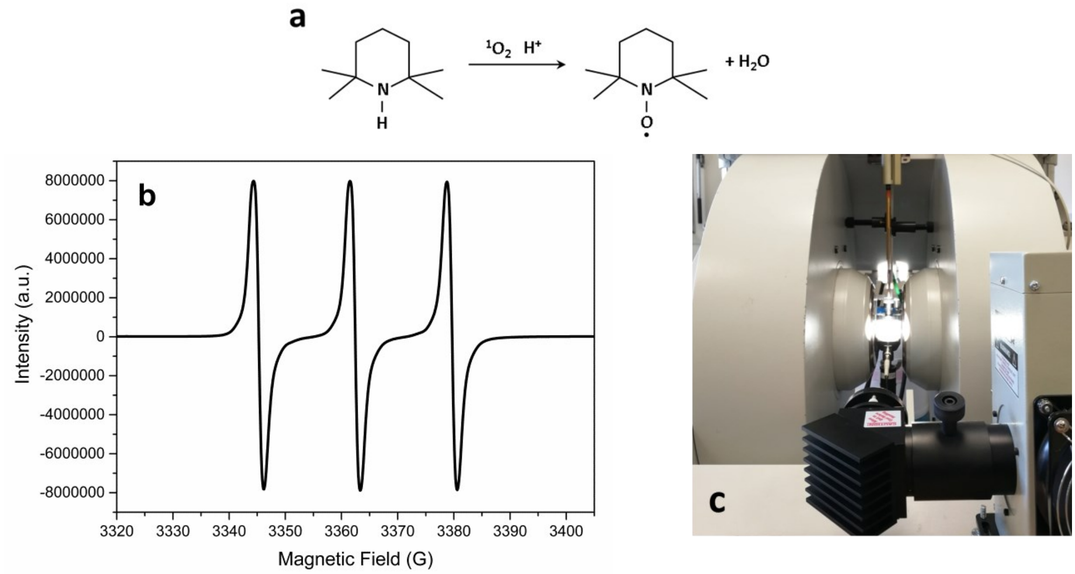

Fig. S3. a) Reaction of TEMP with $^1O_2$ to produce TEMPO **b)** TEMPO EPR spectrum and **c)** EPR equipment.

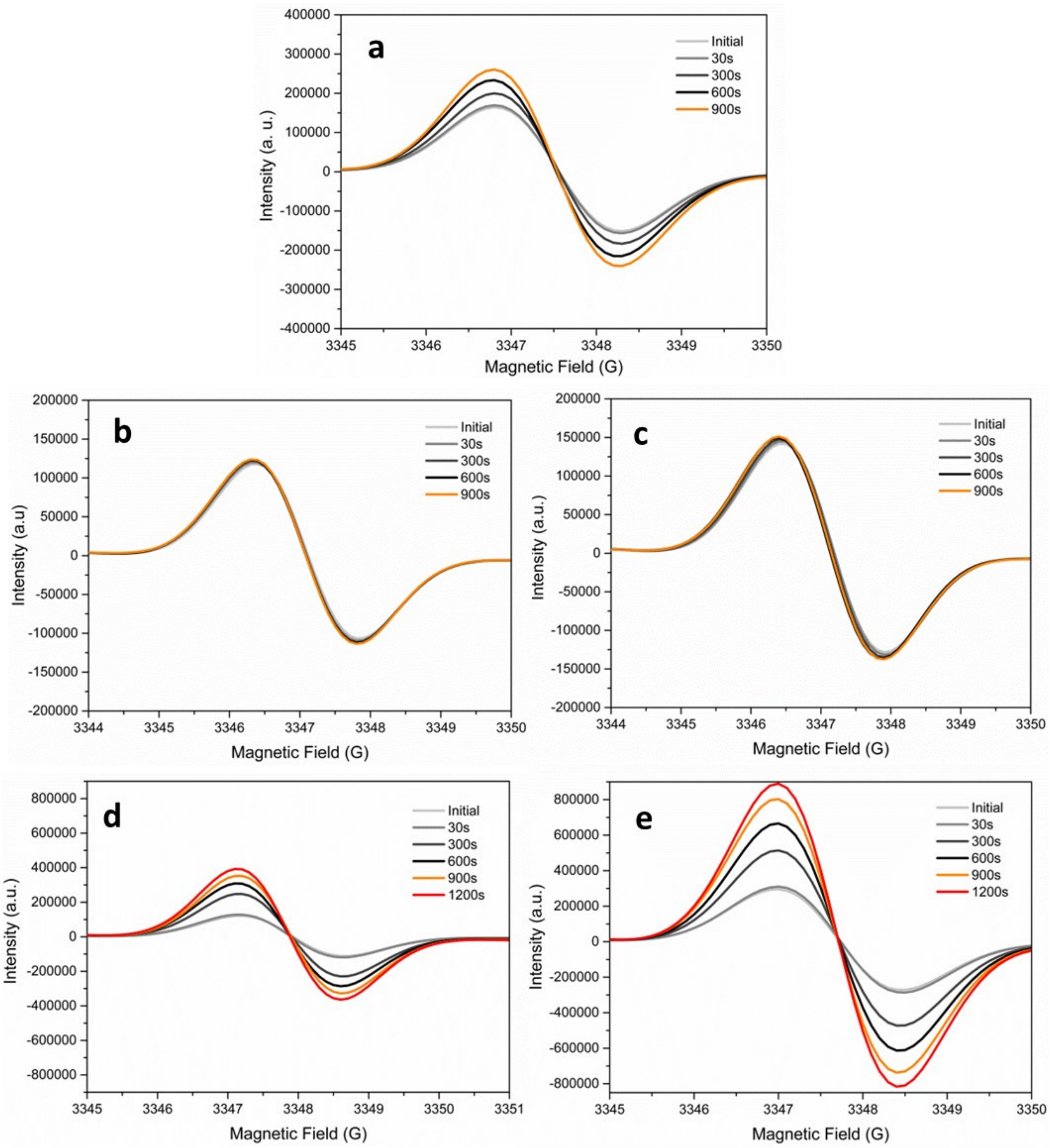

Fig. S4. EPR spectra as a function of time for **a)** Free RB, **b)** AuBPs, **c)** AuBPs@mSiO$_2$, **d)** AuBPs in presence of RB and **e)** AuBPs@mSiO$_2$ in presence of RB.

3. $^1O_2$ generation by oxidation of ADPA

Fitting of the data to a first order kinetic (ln I_0/I vs. t) resulted in a straight line, corresponding to a first order process in respect with the anthracene following the general kinetic equation:

[1] $$d[DPAA]/dt = k_r [^1O_2][DPAA]$$

Where k_r corresponds to the kinetic constant associated to the reaction of singlet oxygen with DPAA

Singlet oxygen is produced by reaction of ground-state, triplet oxygen with the PS at its excited state, following the equation (for a given PS):

[2] $$d [^1O_2]/dt = k_{pn} [PS_n^*][^3O_2]$$

With $[PS_n]$ and k_{pn} being the concentration of photosensitizer n and its photosensitizing kinetic constant, respectively.

The equation of singlet oxygen decay can be written as $-d[^1O_2]/dt = k_{add} [^1O_2][DPAA] + (k_{nr}+k_r)[^1O_2])$, k_r and k_{nr} being the radiative and nonradiative rates of excited (singlet) oxygen in water. Assuming that the monomolecular nonradiative and radiative phenomena of singlet oxygen deactivation are very fast as compared to their generation and reactivity towards anthracene rates, and that singlet oxygene generation is slow in regard with its deactivation, the quasi stationary state approximation can be considered and we can write that

[3]

$$[^1O_2] = \frac{k_{pn}[PS_n^*][^3O_2]}{k_r + k_{nr}}$$

By combining [3] with [1], and by integrating the resulting equation it comes that

[4] $$ln\frac{[DPAA]_0}{[DPAA]_t} = \frac{k_{pn}[PS_n^*][^3O_2]}{k_r + k_{nr}}t$$

Which can be rewritten, defining $K_n = \frac{k_{pn}[PS_n^*][^3O_2]}{k_r + k_{nr}}$

[5]

$$ln\frac{[DPAA]_0}{[DPAA]_t} = K_n t$$

K_n can thus be calculated by linear regression $ln\frac{[DPAA]_0}{[DPAA]_t}$ vs t

For two PS, P_1 and PS_2 associated to a photosensitization kinetic constant k_{p1} and k_{p2} and an apparent constant K_1 and K_2, the ratio of the slopes K_1 over K_2 can be expressed, after simplification of recurring terms by

[6] $$\frac{K_1}{K_2} = \frac{k_{p1}[PS_1^*]}{k_{p2}[PS_2^*]}$$

Now, considering that the solution concentrations have been adjusted so that their absorbance at the irradiation wavelength is constant it comes by definition that $[PS_1^*] = [PS_2^*]$

And the ratio of the slopes $\frac{K_1}{K_2} = \frac{k_{p1}}{k_{p2}} = \frac{\varphi_{\Delta 1}}{\varphi_{\Delta 2}}$

This relation is used here to compare the photosensitization efficiency of all studied PS

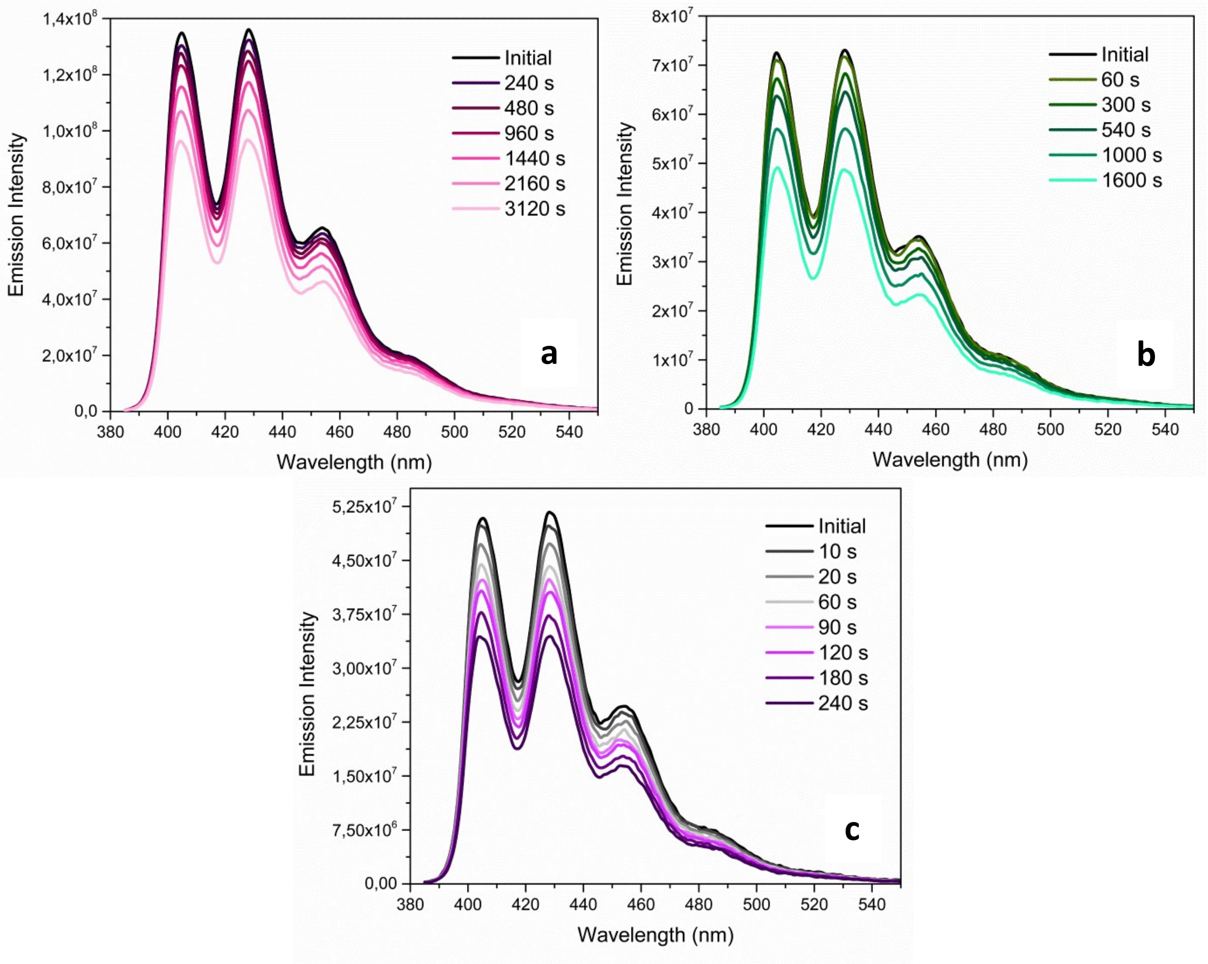

Fig. S5. Emission spectra of ADPA for the samples **a)** RB **b)** AuBPs in presence of RB and **c)** AuBPs@mSiO$_2$ in presence of RB

4. $^1O_2$ photooxygenations in microfluidic reactor

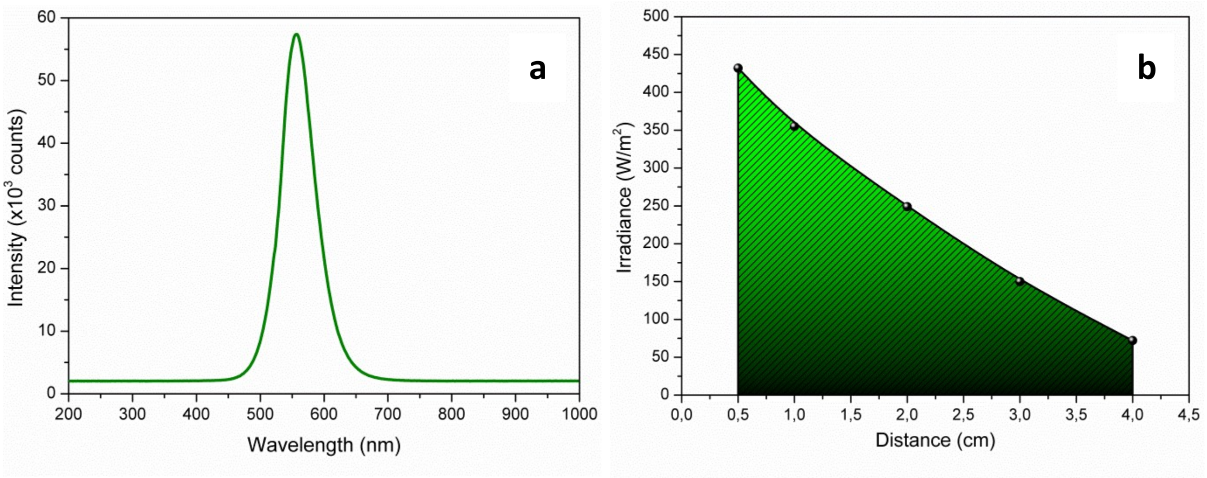

Fig. S6. a) Emission spectrum of the green LEDs b) Irradiance of the green LEDs as a function of the distance

The residence time is calculated according to:

$$Residence\ time\ (min) = \frac{Internal\ volume\ (mL)}{Flow\ rate\ (mL \cdot min^{-1})}$$

The total flow rate combines the individual flow rates of all fluids fed into the reactor. The actual gas flow rate is calculated from the flow rate measured by the MFC according to the following equations:

$$n_{O_2} = \frac{P_N\ (atm) \cdot V_N(L)}{R(L \cdot atm \cdot mol^{-1} \cdot K^{-1}) \cdot T_N(K)}$$

$$V_{real} = \frac{n_{O_2} \cdot R \cdot T_{real}}{P_{real}}$$

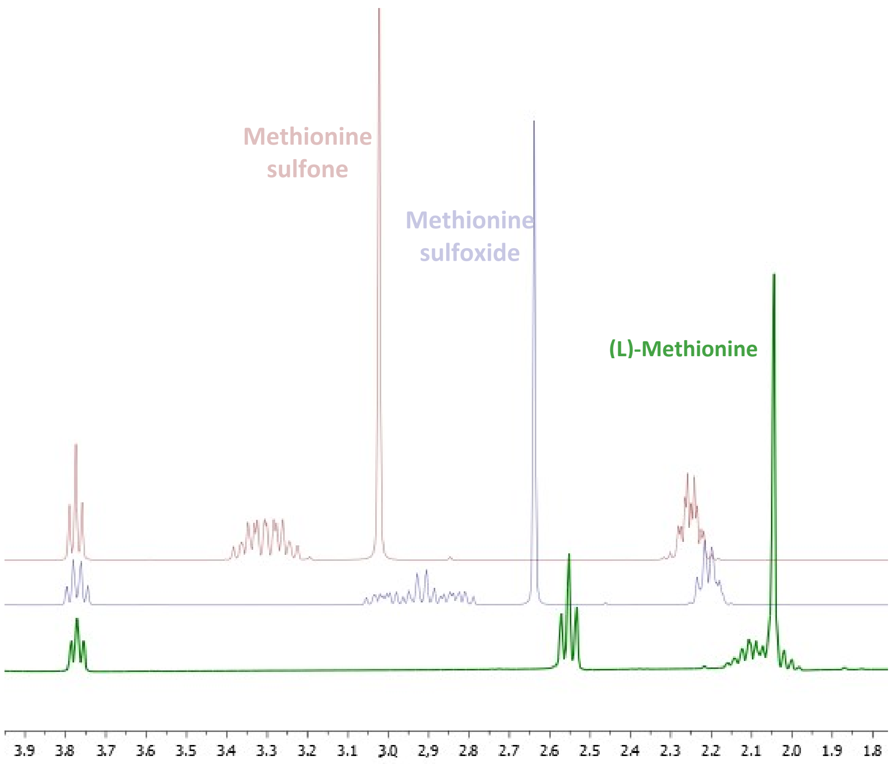

Fig. S7. NMR spectra of Met, MetO and overoxidated methionine sulfone

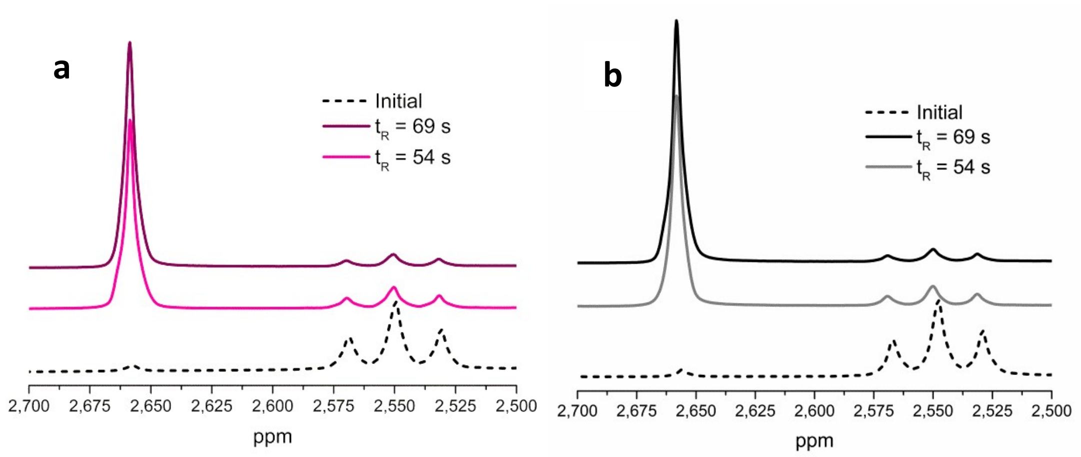

Fig. S8. **a)** NMR spectrum of the initial solution of RB with 0.1M of Met and the obtained NMR spectra after 54 s and 69 s of residence time **b)** NMR spectrum of the initial solution of AuBPs@mSiO_2 in presence of RB with 0.1M of Met and the obtained NMR spectra after 54 s and 69 s of residence time

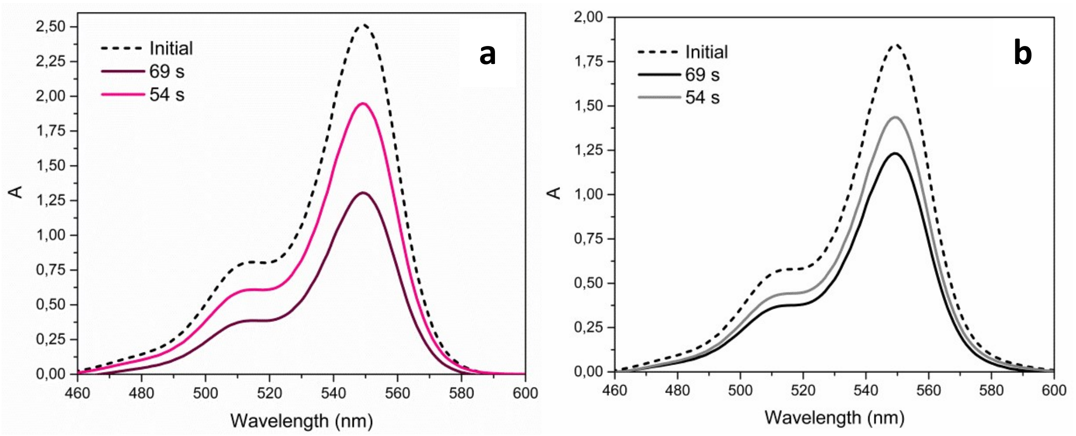

Fig. S9. **a)** UV-Vis spectrum of the initial solution of RB with 0.1M of Met and the obtained UV-Vis spectra after 54 s and 69 s of residence time **b)** UV-Vis spectrum of the initial solution of AuBPs@mSiO_2 in presence of RB with 0.1M of Met and the obtained UV-Vis spectra after 54 s and 69 s of residence time
